# Applying a Design Thinking × COM-B Hybrid Framework to MOW Volunteer Recruitment & Retention

**DOI:** 10.1093/geroni/igaf122.3708

**Published:** 2025-12-31

**Authors:** Ziqi Ma, Cecilia Wang

**Affiliations:** University of Minnesota, Twin Cities, Minneapolis, Minnesota, United States; University of Minnesota Twin Cities, St Paul, Minnesota, United States

## Abstract

This study explores the challenges faced by Meals on Wheels (MOW), including an aging volunteer base, low youth participation, and unstable retention rates, and analyzes how these issues impact service quality and customer satisfaction. To address these challenges, we propose an embedded framework that combines design thinking (empathy, define, ideate, prototype, test) with the COM-B behavior change model (capacity, opportunity, motivation), ensuring that each ideation phase aligns with behavioral drivers. This study employs a mixed-methods approach: quantitative metrics include monthly attendance rates, retention rates at 6 and 12 months, route coverage, and time to fill vacant positions; qualitative data is derived from semi-structured interviews, participant observation, post-intervention surveys, and field notes. Participants included existing volunteers, new volunteers, volunteers who had left within the past year, and project coordinators, ensuring diversity in age, gender, years of service, and route type. Evaluation metrics are used to measure capacity building, changes in motivation, improvements in opportunities, and recurring themes in volunteer feedback. Expected outcomes include increased volunteer numbers, improved recruitment efficiency, improved 6-month and 12-month retention rates, enhanced satisfaction, increased route coverage and reduced last-minute cancellations, and better alignment between volunteer availability and organizational needs. Limitations of the study include geographical constraints, cultural differences, time sensitivity, and self-selection bias. Overall, this framework provides a replicable approach for resource-constrained nonprofit organizations to enhance volunteer recruitment and retention while improving the customer experience.

